# Criticality of plasma membrane lipids reflects activation state of macrophage cells

**DOI:** 10.1098/rsif.2019.0803

**Published:** 2020-02-05

**Authors:** Eugenia Cammarota, Chiara Soriani, Raphaelle Taub, Fiona Morgan, Jiro Sakai, Sarah L. Veatch, Clare E. Bryant, Pietro Cicuta

**Affiliations:** 1 Cavendish Laboratory, University of Cambridge, JJ Thomson Avenue, Cambridge CB3 0HE, UK; 2 Alembic, Experimental Imaging Center, San Raffaele Scientific Institute, Milan, Italy; 3 Department of Veterinary Medicine, University of Cambridge, Cambridge CB3 0ES, UK; 4 Biophysics Department, University of Michigan, Ann Arbor, MI 48109, USA

**Keywords:** liquid–liquid phase separation, plasma membrane composition, macrophage activation, critical lipidomics

## Abstract

Signalling is of particular importance in immune cells, and upstream in the signalling pathway many membrane receptors are functional only as complexes, co-locating with particular lipid species. Work over the last 15 years has shown that plasma membrane lipid composition is close to a critical point of phase separation, with evidence that cells adapt their composition in ways that alter the proximity to this thermodynamic point. Macrophage cells are a key component of the innate immune system, are responsive to infections and regulate the local state of inflammation. We investigate changes in the plasma membrane’s proximity to the critical point as a response to stimulation by various pro- and anti-inflammatory agents. Pro-inflammatory (interferon *γ*, Kdo 2-Lipid A, lipopolysaccharide) perturbations induce an increase in the transition temperature of giant plasma membrane vesicles; anti-inflammatory interleukin 4 has the opposite effect. These changes recapitulate complex plasma membrane composition changes, and are consistent with lipid criticality playing a master regulatory role: being closer to critical conditions increases membrane protein activity.

## 1. Introduction

Macrophages are extremely versatile cells of the innate immune system that are able to activate and adapt their functionality depending on the specific milieu [1]. Following phagocytosis of material resulting from trauma, or pathogens, or detection of specific functional molecules, macrophages can change their gene regulatory state and polarize into activated states, where, for example, they produce immune effector molecules such as cytokines for intercellular communication [2–4]. The responses manifested as a consequence of different types of stimulation have been classified into two broad activation states, based on both genetic expression profiling and phenotypic behaviour: M1, or classically activated, macrophages have an enhanced bactericidal and tumoricidal capacity and produce high levels of pro-inflammatory cytokines, while M2 macrophages produce low levels of cytokines and have a wound-healing capacity by contributing to the production of collagen and extracellular matrix [1,3,5]. The stimuli that promote M1 macrophage activation are mainly interferon *γ* (IFN-*γ*), lipopolysaccharides (LPSs) and granulocyte–macrophage colony-stimulating factor (GM-CSF). IFN-*γ* is a cytokine mainly produced by natural killer (NK) and T helper 1 (Th1) cells; signalling from the IFN-*γ* receptor (IFNGR) controls the regulation of specific genes related to the production of cytokine receptors, cell activation markers and adhesion molecules [1]. LPSs are a class of molecules of the outer membrane of Gram-negative bacteria; these molecules are recognized by the TLR4 receptor [6,7]. TLR4 activation triggers the downstream production of pro-inflammatory cytokines such as tumour necrosis factor α (TNF-α) and presentation of antigens [8]. By contrast, macrophages polarize into M2 mainly in response to interleukin 4 (IL-4) and IL-13 stimuli. IL-4 is produced by T helper 2 (Th2) cells, basophils and mast cells in response to a tissue injury and in the presence of some fungi and parasites [3]. M2 cells are sensitive to infections, their production of pro-inflammatory cytokines is minimal and their phagocytic activity is low [1,3].

**Table 1.** Summary of the numerical values of the miscibility temperature and transition width obtained by fitting the data with the empirical function *f*(*T*) = *A* [tanh ((*T* − *T*_*m*_)/*σ*) + 1] + *C*.

stimulation		*T*_*m*_ (°C)	*T*_*m*_ err (°C)	*σ* (K)	*σ* err (K)
IL-4	12 h	12.42	0.98	3.28	0.99
	12 h	12.42	0.79	3.19	0.81
	24 h	10.46	0.33	3.35	0.35
	24 h	14.54	1.06	5.46	1.56
unstimulated		18.91	0.89	5.45	1.57
		14.56	0.80	5.75	1.24
		15.88	0.46	4.39	0.61
		18.18	0.43	4.17	0.56
		14.00	0.69	5.20	0.99
		13.11	0.49	4.90	0.62
		15.88	0.46	4.39	0.61
		16.44	0.83	4.28	1.03
		14.82	0.70	4.23	1.31
		14.21	1.02	5.31	1.44
		16.95	0.50	6.79	0.85
		16.11	0.60	8.25	1.12
IFN 12 h		20.38	0.99	6.79	1.97
		19.04	0.87	6.73	1.51
		18.81	0.45	5.09	0.78
		22.09	0.21	1.62	0.32
LPS 12 h		20.15	0.20	3.84	0.30
		18.77	0.89	7.12	1.63
		18.36	0.61	4.84	1.03
		15.74	0.93	7.50	1.59
		21.33	0.41	4.95	0.72
		16.35	0.74	6.27	1.51
KLA 12 h		15.93	0.88	6.35	1.43
		25.93	0.90	6.02	1.68
		15.63	1.00	7.60	1.70
TLR4^−/−^ unstimulated		16.48	0.68	4.22	1.06
TLR4^−/−^ KLA		15.43	0.93	4.84	1.38

In the transduction of signals a fundamental regulatory role is thought to be played by the plasma membrane composition [9]. There are many examples of specific protein–lipid affinity, but also strong evidence of more general mechanisms such as the propensity of lipid mixtures to form cholesterol-rich domains, or domains of a preferred thickness, which then imply a preferred partitioning of certain transmembrane proteins [10–12]. Any mechanism that modifies local recruitment of membrane proteins, in the context of an assembly step such as dimerization, which is necessary for function, can therefore directly be a regulator of receptor activity. This generalizes a well-known theme in membrane biochemistry: that proteins with lipid raft affinity have a higher chance to interact [13]. The key structures in this study of macrophages, the TLR4 receptor and its co-receptor CD14, are both known to have raft affinity: CD14 is found in lipid rafts both before and after LPS activation, while TLR4 receptors are initially found in non-raft regions and then translocate to rafts after the activation [14]. It has also been shown that the use of lipid raft inhibitors reduces significantly the production of cytokines related to LPS activation [15]. Moreover, lauric fatty acid seems to be responsible for the recruitment and dimerization of TLR4 into lipid rafts [16]. All together these facts strongly hint that plasma membrane composition and, in particular, the propensity to form lipid rafts or domains are fundamental regulators of protein interaction; we explore this theme with respect to activation of macrophages and the activity of TLR4 receptors.

Various authors have put forward the idea that the lipid raft phenomenology is linked to the propensity for the lipidic component of the membrane to undergo liquid–liquid phase separation [12], as was observed in plasma membrane extracts [17]. Vesicles extracted from the plasma membrane of cells have the same characteristics as certain ternary lipid mixtures; of particular interest is the spontaneous appearance of transient lipid domains, which is a universal property of systems in the vicinity of a critical point [17,18]. From a biological point of view, being poised close to a critical point could be advantageous to accelerate a whole set of membrane biochemistry, since the cell would require much less energy to create lipid heterogeneity. Modulating the lipid composition is thus a mechanism for global regulation of activity on the membrane [12]. Giant plasma membrane vesicles (GPMVs) allow the properties of membrane lipids to be studied as isolated systems [19,20]. These vesicles are thought to maintain the protein and lipid diversity of the mother membrane [20,21], and at low temperatures the lipids can phase separate laterally into micrometre-sized domains [17,22,23].

GPMVs, as systems to study the transition temperature of the plasma membrane, have shown systematic dependency on growth, temperature [24] and cell cycle [25], and on the epithelial–mesenchymal transition in cancer cells [26]; indeed, in both situations the transition temperature of GPMVs recapitulates broad systematic composition changes that move the cell composition closer to or further from the critical point. In the literature, there are previous studies on the effect on lipid composition of macrophage activation [27,28], but these are bulk assays and report on the changes in a huge number of lipid species, making it difficult to interpret the results in simple terms. The work presented here shows that these complex changes in lipidomics, as reported in the literature [27,28], may have a simple interpretation in terms of their effect on the membrane phase separation. Investigating the effects of different kinds of macrophage cell stimulants (LPS, Kdo 2-Lipid A (KLA), IFN-*γ*, IL-4) known to differentiate macrophages into two different activation states, we show opposite changes with respect to the proximity of the critical point in the two cell types, consistent with biological function.

## 2. Material and methods

### 2.1. Cell culture

The immortalized BMDM cell lines were obtained from Dr Eicke Latz (Institute of Innate Immunity at the University of Bonn, Bonn, Germany) and Dr Kate Fitzgerald and Dr Douglas T. Golenbock (University of Massachusetts Medical School, MA, USA). C57BL6 TLR4^−/−^ mice were obtained from Dr S. Akira (Osaka University, Osaka, Japan) [29]. iBMDM and TLR4^−/−^ iBMDM were maintained in Dulbecco’s modified Eagle’s medium (Sigma-Aldrich, MO, USA) supplemented with 10% (v/v) heat-inactivated HyClone fetal calf serum (Thermo Scientific, UT, USA), 2 mM L-glutamine (Sigma-Aldrich), 100 U ml^−1^ penicillin and streptomycin (Sigma-Aldrich), and 20 mM HEPES (Sigma-Aldrich). Cells are cultured for at least 2 days and brought to confluence in a single 175 cm^2^ flask. From confluence, cells are plated in separate dishes. To test the effect of stimulants on the melting temperature an equal number of cells are plated for each condition; we use a density of about 6 − 7 × 10^3^ cells mm^−2^. After 12 h, the culture medium is changed with (or without for the control condition) the addition of stimulating agents. Then, after the stimulation time, we start the GPMV production protocol. Cell-stimulating agents are used at the following concentrations and with the following timings: IFN-*γ* 20 ng ml^−1^ (PeproTech) for 12 h; LPS from *Salmonella* Typhimurium 10 ng ml^−1^ (Enzo Life Sciences) for 12 h; KLA 100 ng ml^−1^ (Avanti Polar Lipids) for 12 h; IL-4 20 ng ml^−1^ (PeproTech) for 12/24 h. These doses where chosen according to previous work on M1/M2 macrophage differentiation [30–32].

To measure the transition temperature (*T*_*m*_) versus cell density dependency, density was measured in two different ways. For some experiments, images of the culture were acquired with a low-magnification objective and the density estimated by counting cells from the image and then dividing their number by the field-of-view area. The same dish was then used to produce GPMVs immediately after. Otherwise for each density we had twin dishes: one was used to count the cells with the haemocytometer, while the other was used to produce GPMVs. To check the effect of stimulation on growth rate, an equal number of cells were plated in a multi-well for each condition (control, IL-4, LPS); cells where counted with the haemocytometer after cell adhesion (0 h), then stimulated, according to previously specified concentrations, and counted after 12 h. Cells were initially plated to have about 6 − 7 × 10^3^ cells mm^−2^ at 0 h.

### 2.2. GPMV production

The procedure for membrane labelling and GPMV production follows the protocols in [33,34]. The cells are gently washed twice with phosphate-buffered saline (PBS); then DiI-C12(3) (Life Technologies) dye solution 50 μg ml^−1^ in PBS is added and left on ice for 10 min to allow incorporation into the membrane. Then the cells are washed five times with PBS and twice with GPMV buffer. GPMV buffer consists of 10 mM HEPES, 150 mM NaCl, 2 mM CaCl_2_, and the pH is adjusted to 7.4 with HCl or NaOH. Lastly, the vesiculating agent is added and the cells are left in the incubator for 1.5 h at 37°C; 20 μl of vesiculating agent (2 mM dithiothreitol (DTT), 25 mM paraformaldehyde (PFA)) is used for each ml of GPMV buffer. The medium is gently harvested and transferred into a falcon tube. The sample is left at 37°C for long enough to let the blebs deposit on the bottom of the tube: for a volume of 4 ml, 24 h are enough for the whole sample to sediment.

### 2.3. Isolation of lipids and gel-assisted vesicle formation

For the lipid isolation procedure, we followed the Bligh and Dyer method [35]: 1 ml of GPMV sample is collected and moved to a vial. Then 3.75 ml of a 1:2 chloroform and methanol mixture, 1.25 ml of chloroform and 1.25 ml of distilled water are added. After each step, the solution is vortexed for 1 min. At this stage, the GPMVs burst and the components dissolve in the solution. The mixture is then centrifuged at 1000 r.p.m. for 5 min. This makes the chloroform/methanol fraction deposit at the bottom of the tube, together with the lipids, while the aqueous and water-soluble components are isolated at the top. Proteins are preferentially located at the interface between the two phases. The bottom phase is then collected and left under vacuum to let the solvents evaporate. Finally, lipids are re-dissolved in 100 μl of chloroform.

The vesicles are produced through the gel-assisted method as described in [36]: 200 μl of 5% (weight/weight) polyvinyl acetate (PVA) solution is spread on a microscope coverslip with the help of a spincoater and then left to dry in an oven at 50°C for 30 min. Lipids dissolved in 100 μl of chloroform are then spread on PVA gel. A chamber is formed with the help of a spacer and a second coverslip and filled with a solution of sucrose. After 30 min, the vesicles are collected and diluted in glucose solution to allow vesicle sedimentation.

### 2.4. Imaging

The samples are imaged on a Nikon Eclipse Ti-E inverted epifluorescence microscope using a Nikon PLAN APO 40 × 0.95 N.A. dry objective and an IIDC Point Grey Research Grasshopper-3 camera. The perfect focus system (Nikon) maintains the sample in focus even during thermal shifts. The temperature of the sample is controlled with a home-made computer-controlled Peltier device. A thermocouple is placed in direct contact with the sample chamber. In each position a *z*-stack of eight images is acquired, spanning across a range similar to the bleb size. The temperature is decreased across the whole sample with a ramp from 37°C to 3°C in steps of 2°C; at each step, the temperature is allowed to equilibrate for 15 s. The abundance of GPMVs produced could vary from cells prepared on different days, but usually from a dish of 5.5 cm diameter with confluent cells it is possible to produce blebs for at least two experiments. With the quantities described above, we are able to image up to 100–200 blebs in each field of view.

### 2.5. Software processing

A custom Matlab software pipeline was developed to automatically detect the position and radius of the GPMVs in the images. It uses the Hough transform to detect circular features. Then with the help of a graphical user interface the blebs are shown to the user one at the time; the user can interactively scroll the *z*-stack and decide if the bleb shows (a) a single phase, (b) phase coexistence, or (c) unclear phenotype. The software randomly picks the vesicle to show, from the database of all the temperatures, i.e. in this stage the information about the temperature is kept hidden from the user, so that the decision process (assigning the type a/b/c) is unbiased.

## 3. Results

**Figure 1.**
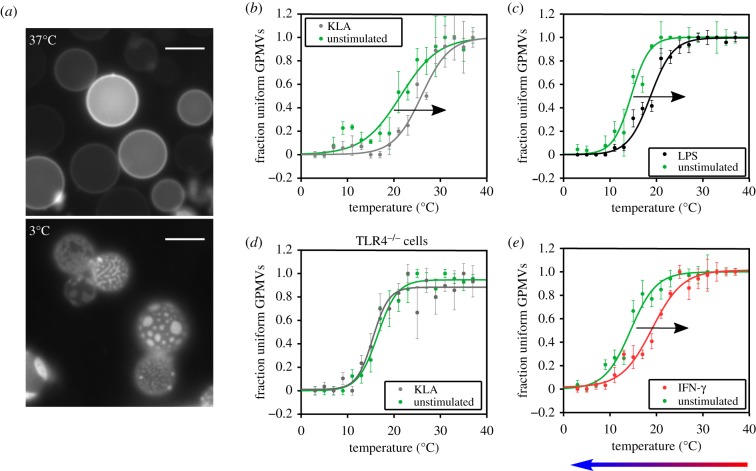
The plasma membrane of macrophage cells is close to critical composition and changes its transition temperature in response to signalling molecules. (*a*) Fluorescence microscope image of GPMVs at 37°C and 3°C. Scale bar, 5 μm. (*b*–*e*) Fraction of GPMVs showing just one phase over the total of vesicles observed as a function of the temperature. The data show a sigmoidal trend and are fitted with a hyperbolic tangent from which are extracted the transition temperature at mid-height and the width of the transition. We compare the samples obtained from cells treated with KLA (*b*), LPS (*c*) and IFN-*γ* (*e*), for 12 h, with a non-treated control condition prepared in parallel. All these ‘pro-inflammatory’ treatments shift the transition temperature towards higher temperatures. The coloured arrow at the bottom indicates the direction of the temperature variation imposed on the GPMV samples during the imaging process. (*d*) The knock-out TLR4^−/−^ cells do not vary the transition temperature when stimulated with KLA (in contrast to (*a*)), remaining the same as the unstimulated controls. (Online version in colour.)

Following established protocols, GPMVs are produced from macrophages using PFA and DTT. The sample is observed with an optical microscope on a temperature-controlled stage. The temperature is lowered from 37°C to 3°C in steps of 2°C. At high temperatures, all the vesicles show a uniform phase. At around 12–22°C phase separation domains start to appear in some GPMVs, and at low temperatures most of the GPMVs are phase separated (figure 1*a*). Approaching the transition temperature from below, the contours of the phase separation domains are increasingly less smooth, and become progressively rough and fragmented until the two phases are completely mixed (see electronic supplementary material, figure S1). Similar image sequences were shown in papers where other aspects of criticality were tested directly [17]. This morphology of domains with temperature suggests that the GPMVs from macrophages have compositions close to critical. For each temperature, we calculate the fraction *f*(*T*) of GPMVs which show uniform phase or phase separation. Before producing GPMVs, macrophages are stimulated with one of IFN, LPS or KLA for 12 h to induce a pro-inflammatory response. The effects of LPS stimulation on iBMDMs has been tested and characterized in detail in the NF-κB (nuclear factor kappa-light-chain-enhancer of activated B cells) pathway in our previous study [37], where cell lines with fluorescent markers were used to measure NF-κB translocation, and TNF-α promoter activation as a downstream effect. In each dataset (figure 1*b*–*e*), we compare the stimulated condition with its unstimulated control dataset, since we noted (as has been already reported in different cell types [24,34]) a significant variability in the transition temperature of independent repeats; by contrast, the transition temperatures of GPMVs from the same cultures, even split into separate dishes, are tightly distributed.

The transition temperature *T*_*m*_ is obtained by fitting the *f*(*T*) data with an empirical sigmoidal curve

3.1 $$f(T)=A\left[\tanh\left(\frac{T-T_{m}}{\sigma }\right)+1\right]+C,$$

where *T*_*m*_ and *σ* are the most interesting parameters to describe the mean and the cell-to-cell variability (GPMVs originate from individual cells) in the transition temperature of the population. Error bars are associated with data points by randomly separating the measurements for a given temperature into three groups, and treating these as independent datasets. Table 1 summarizes all data fitted with equation (3.1).

Figure 1*b* shows the effect of the cell stimulation with Kdo 2-Lipid A (KLA) for 12 h. The comparison with the control condition shows a shift of 4.5°C in the GPMV transition temperature to higher temperatures. As expected, LPS and KLA stimulation produce similar effects (figure 1*b*,*c*). Indeed KLA is the active subunit of the LPS molecule which is recognized by the TLR4 transmembrane receptor [6]. Note that the comparison between LPS and KLA has to remain qualitative since there is not a first-principles way to correlate the doses, except for the effects on activating cells. Both doses employed here are known to be able to saturate the cell response, for example in terms of TNF-α production [28,37,38]. As a control, repeating the same experiment of KLA stimulation, this time on TLR4^−/−^ macrophage cells, we obtained compatible transition trends (figure 1*d*) between the stimulated and unstimulated conditions. The absence of a lipid change in the cells without receptors strongly suggests that the observed phase transition temperature shift originates from the metabolic change as a downstream effect, and not from a direct membrane perturbation by the ligand. We then stimulated cells with IFN-*γ*, which is known, like LPS, to have pro-inflammatory effects [1], and obtained the same qualitative effect on the plasma membrane transition temperature (figure 1*e*).

**Figure 2.**
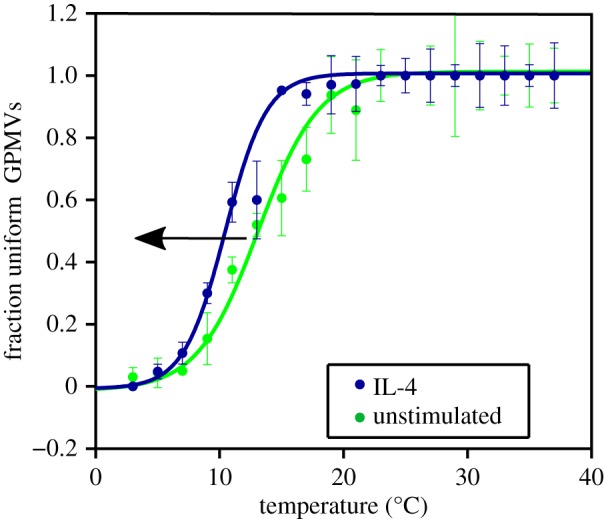
Anti-inflammatory treatment changes the melting temperature in the opposite direction compared with pro-inflammatory stimuli, consistent with changes in the composition of the membrane away from the critical point. The data show the fraction of uniform GPMVs as the temperature of the sample is varied. The two curves correspond to 24 h of IL-4 stimulation and to unstimulated conditions. *T*_UNST_ = (13.11 ± 0.49)°C, *T*_IL4_ = (10.46 ± 0.33)°C. (Online version in colour.)

Since all the experiments with the ‘classically activated’ conditions showed a consistent shift in the same direction, we decided to stimulate the cells with IL-4, which is known to induce a different type of differentiation [3]. Macrophages treated with IL-4 have different phenotypes and markers compared with M1, and a different role in the immune response: they do not produce pro-inflammatory cytokines, but suppress destructive immunity, and are involved, for example, in the wound-healing response [3]. IL-4 is also known to induce polarization in the same cell line (iBMDM) [39]. The curves in figure 2 correspond to the control condition and to 24 h of IL-4 stimulation. Also, in this case, the stimulation produces a temperature shift, but in contrast to the ‘classically activated’ cells the *T*_*m*_ shifts towards lower temperatures.

**Figure 3.**
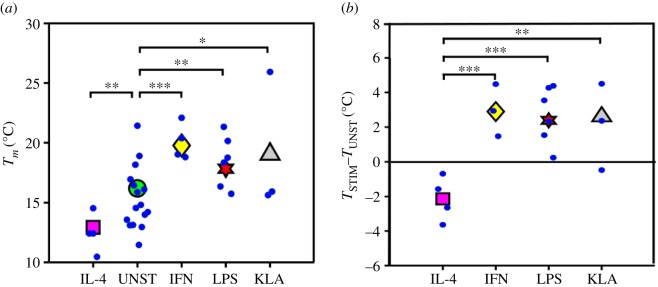
Pro- and anti-inflammatory treatments affect the transition temperature systematically. The scatter in the absolute transition temperature (particularly notable in the unstimulated (UNST) cells) is reduced significantly compared with same-day unstimulated controls. (*a*) Fitted transition temperatures of vesicles produced by macrophage cells treated with IL-4, IFN-*γ*, LPS or KLA. Each small data marker comes from an experiment with between 300 and 600 vesicles. The large markers indicate the average in each distribution, weighted with the errors on *T*_*m*_. (*b*) Temperature difference of each stimulation experiment with its control condition. From one-way analysis of variance, we obtained the distribution differences to be statistically significant with *p < 0.1, **p < 0.05, ***p < 0.005. (Online version in colour.)

Collecting together all the *T*_*m*_ values from different stimulation experiments (table 1; figure 3*a*) we can see how the IL-4 data and the IFN-*γ*/LPS/KLA data are in two separate temperature ranges, with no data overlapping, while the values from the unstimulated experiments have a much wider range. Statistical analysis confirms the distributions to be significantly different (*p* < 0.05) for almost all of the conditions. Calculating the temperature differences *T*_STIM_ − *T*_UNST_ (i.e. comparing with same-day controls), the temperature shifts tighten (figure 3*b*) and show very consistent behaviours: the IL-4 data points are all negative, whereas the others are all positive. Similar temperature shifts, of about 2–4°C, have also been found when comparing melting temperatures of GPMVs from human mesenchymal stem cells differentiated into osteoblasts or adipocytes. Also, in this case, the plasma membrane lipidic composition is thought to play a key role into tuning lineage specification [40].

We then investigated cell density as one of the possible causes for the large variability of *T*_*m*_ in the control condition. The effectiveness of intracellular communication indeed depends on the cell density, and can be conveyed through both mechanical and chemical interactions [41–43]. A set of careful experiments (see electronic supplementary material, figure S2) shows a linear trend of the miscibility temperature as a function of the cell density, with the higher densities inducing a shift in the *T*_*m*_ in the same direction as the IL-4-activated samples. A similar density effect has been observed in similar experimental conditions for other cell types [24]. We also found that LPS stimulation induces differences in the population growth rate, which complicates comparison between controls if one wants the cell density to match. Putting together the cell density assay with the measurement of cell growth, we found that with our cell growth protocol the difference in densities between treated and control conditions can lead to an effect on only about 0.5°C of the 2°C shifts measured for *T*_*m*_ (see electronic supplementary material, figure S2*b*).

**Figure 4.**
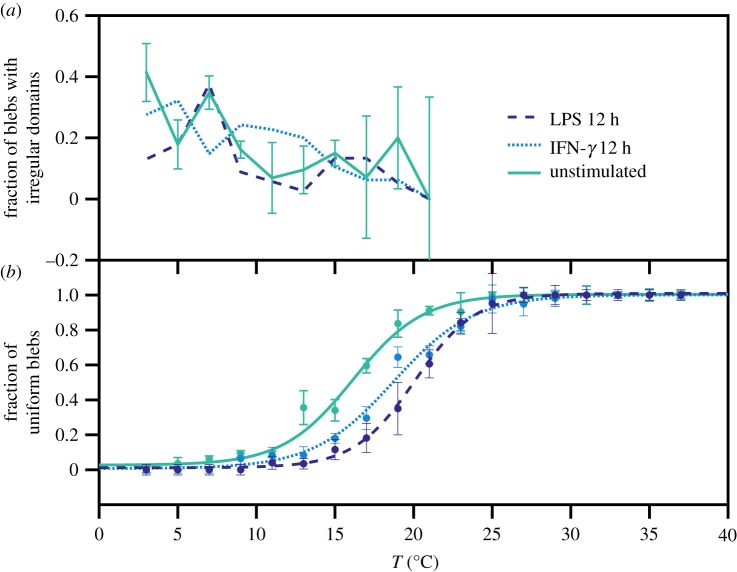
At very low temperatures, irregular-shaped domains are observed and attributed to a gel phase. (*a*) The fraction of GPMVs with irregular domains (over the total of phase-separated GPMVs) increases at low temperatures. This fraction grows below *T*_*m*_, as can be seen by comparing in (*b*) the ‘conventional’ data on liquid–liquid phase separation for the three conditions indicated in the legend. See microscopy images in electronic supplementary material, figure S3. (Online version in colour.)

The high-quality imaging also allowed us to investigate the shape of the phase separation domains appearing in blebs at low temperatures. Some of the domains indeed appear to have an irregular rigid shape similar to a gel phase domain, while others look more rounded, such as in the liquid–liquid coexistence. With the help of a graphical user interface that shows three- or four-frame time sequence of the vesicle, GPMVs with irregular domains were identified as those presenting rigid and not rounded dark regions (see electronic supplementary material, figure S3). The appearance of gel-type domains on GPMVs has already been reported [24], but this is the first attempt to quantify the phenomenon. Three sets of data under different conditions are shown in figure 4. In all the cases, in spite of the noise, the fraction of irregular domains over the total of phase-separated GPMVs has a clear growth at low temperatures, reaching about 0.4 at 3°C. On the other hand, we do not see any significant difference in these trends when comparing different stimulation conditions, suggesting that they might not play a critical role in cell activation and differentiation. In the event that these irregular domains could be confirmed as gel domains, this kind of analysis would provide an additional piece of information on the phase diagram of the biological membrane lipid mixture (on which we still have very incomplete knowledge) and might be particularly important in cell biology regulation involving cholesterol [44].

The experiments described so far provide evidence that the composition of the plasma membrane is regulated according to the external milieu, but we still do not know if the phase separation phenomenon is lipid driven or if the proteins have any role in the formation of lipid compartmentalization. To address this, we performed an important and seldom considered control: comparing the melting temperature of GPMVs with the same sample after a lipid purification process. A similar experiment has been pioneered by Dietrich *et al.* [45]. The GPMV sample was divided into two aliquots, and one of them was dissolved, purified and the vesicles re-formed through a gel-assisted formation technique, as described in the Material and methods section. The purified GPMVs produced with this protocol are very few compared with the control sample; moreover, it is easier to find them clumped together and they are on average smaller. This makes the recognition of phase separation domains more difficult and prone to errors. Nevertheless, as the standard and the purified GPMVs show phase separation at low temperatures as well as a very similar phase transition curve (see electronic supplementary material, figures S4 and S5), this means that the phase separation phenomenon on GPMVs is lipid driven and that the miscibility temperature is mostly unperturbed by membrane proteins. It is worth remarking here that this experiment has to be interpreted as a qualitative result since we do not have proof that the lipid mixture is preserved identically after the purification process; moreover, in the reconstituted vesicles, we would have lost any bilayer asymmetry possibly maintained in the GPMVs.

## 4. Discussion

It is well known that the plasma membrane is not just a passive support for activity by membrane proteins, and here we have developed the theme that the property of lipids to phase segregate relates to protein interactions [12,46]. GPMVs are an extremely useful system to understand this aspect of plasma membranes because they maintain the composition of the original membrane, but they can also be studied as an isolated structure and subjected to stringent controls. Our results add to the body of evidence that proximity to the critical point for phase separation can be a global regulator favouring activity. For the specific case of macrophage cell activation, our results are consistent with previous findings that TLR4 receptor oligomerization takes place in raft domains [14]. Indeed melting temperatures closer to the physiological temperature produce bigger and longer lasting fluctuations in spontaneous domain formation with a higher chance of protein interaction. Oligomerization of TLR4 would be further promoted as a positive feedback loop, as well as promotion of oligomerization of any other membrane receptor that partitions preferentially into lipid domains. These mechanisms would reinforce signalling cascades and the commitment of macrophages to an activated state.

### 4.1. Effect of stimulation on plasma membrane transition temperature

We have seen how treatment of macrophages with different stimulating agents affects the melting temperature of GPMVs. All the stimulants used (IFN-*γ*, LPS, KLA and IL-4) induced a shift of a few degrees compared with the control condition, meaning that in all the cases the membrane composition has changed as a consequence of the activation of specific signalling pathways. Moreover, IFN-*γ*, LPS and KLA increased the transition temperature (*T*_*m*_), whereas IL-4 had the opposite effect—decreasing *T*_*m*_. Given that the first three stimulants can be connected to the activation into the M1 state in macrophages, whereas IL-4 is responsible for the differentiation into the M2 state, this result sheds new light on the importance of plasma membrane composition in the immune response, and suggests new ways in which lipid composition may be involved in the regulation of the host defence strategy.

From the point of view of the membrane composition, if the melting temperature increases (coming closer to physiological temperature) it means that spontaneous lipid domains are longer lived and larger, so that membrane components can partition more strongly; also, the energy cost to recruit a particular lipid micro-environment around a protein is reduced [46]. It has been calculated that because of this universal phenomenon, the proximity to critical point, spontaneous lipid domains exist at sizes of around 22 nm for GPMVs from rat basophilic leukaemia cells (RBL) cells [17]. This argument considers the dimension of the correlation length *ξ* at a physiological temperature (*T* = 37°C) and experiments that measured *T*_*m*_, using the expression *ξ* = *ξ*_*o*_ *T*_*m*_/(*T* − *T*_*m*_) [18]. This same argument can now be extended, in light of the temperature shifts presented here and assuming, as we show from domain morphology, that the composition of GPMVs from macrophages is close to a critical point. Keeping the same value of *ξ*_*o*_ from [17] (because this is a quantity similar to the size of the lipid) we can estimate the effect of an increase in *T*_*m*_ from *T*_*m* M1_ = 13°C to *T*_*m* M2_ = 20°C (from figure 3). This results in an increase in the correlation length of the order of 40% (from *ξ*_M1_ = 12 to *ξ*_M2_ = 17 nm). We expect this to have an effect on the confinement of proteins and their local concentration. In the particular system here, we can expect this to affect the balance of dimerization in membrane receptors (TLR4 itself, hence positive feedback) and hence regulate a variety of signalling pathways that move the macrophage to the committed activate state [47,48]. The arguments on the changing proximity to the critical point are based on the temperature shifts, which are well defined and consistent over the experiments; the absolute temperatures, however, may change with cell density and have a day-to-day variability (see electronic supplementary material, figure S6).

### 4.2. Speculative correlation between membrane composition and receptor activity

We suggest here a possible correlation between the role of the cell in immune defence and the changes in its membrane composition. One can imagine that these cells, depending on their activation state, regulate their lipid composition in such a way as to tune the proximity to the critical point, and hence, in turn, the typical dimension and lifetime of spontaneous lipid domains, in order to be more or less reactive towards external stimuli. An M1 cell would have bigger and more long-lasting lipid domains, leading to increased activity of TLR4 receptors, which have raft affinity [14,49–51] (e.g. by increased recruitment to the membrane, and increased dimerization), to induce a faster and stronger inflammatory response with consequent production of inflammatory cytokines. By contrast, in M2 cells the activation of the TLR4 to NF-κB pathway would be down-regulated through the lipid composition effect. An important element in support of this hypothesis is the reported increased sensitivity to LPS after IFN-*γ* treatment, both in mice [52] and in macrophages in vitro [53], where a 66% increase of the LPS binding efficiency has been measured. In general, activation of TLRs can induce long-term changes in the way a cell responds to further stimulation with TLR ligands including sensitization or tolerance [54].

### 4.3. Effect of cell density on *T*_*m*_

To investigate the cause of the *T*_*m*_ day-to-day variation, the effect of cell density was tested. The results show that denser populations induce a lower *T*_*m*_ in GPMVs. The same experiment has been performed on RBL cells [24] with the same outcome, the authors suggesting that dense populations could have different physical membrane properties to be able to sense and communicate with touching cells [55].

Regarding macrophages, one could relate this result to the shift given by the different kinds of stimulations, venturing a picture in which the overcrowded populations have some common behaviour with M2 cells. Our hypothesis is that cell density indirectly induces a decrease in *T*_*m*_, perhaps by triggering the production of cytokines with the same effect of IL-4. This idea is supported by a study in which M1/M2-like differentiation was induced by the population density [56]. Moreover, BMDMs from high-density cultures secrete less pro-inflammatory cytokines, have lower phagocytic ability, and the number of cells showing typical M2 membrane markers like CD11c and Ly-6CLy-6G increases [56]. In this picture, the crowded populations, with no need to further recruit cells and promote additional inflammation against possible infections, diminish their cytokine production, thus acting more like M2 cells.

To test the hypothesis of the interaction through cytokines, we performed an experiment where the medium was periodically changed every 2 h. The ‘washed’ sample shows a higher *T*_*m*_ than the control, where cytokines would be accumulating in the medium; see electronic supplementary material, figure S7. This is compatible with a scenario in which the control condition is affected by an accumulation of M2-inducing cytokines such as IL-4.

Even though the density has been proven to be an important factor in the day-to-day variability in the *T*_*m*_ of unstimulated macrophages, this is not enough to fully explain the variability between independent repeats; indeed, just keeping the cells in separate cultures is enough to produce some variability (electronic supplementary material, figure S6).

## 5. Conclusion

The biological question addressed here concerns macrophage cells, which we conditioned via pro- and anti-inflammatory stimuli, before extracting GPMVs and measuring their phase transition temperatures. From the morphology of domains, it is clear that phase separation happens in the proximity of a critical point (second-order transition). Considering all the transition temperatures together, we get a very consistent picture: transition temperatures following IL-4, as opposed to IFN-*γ*/LPS/KLA treatment, form two non-overlapping intervals (respectively, at 10–15°C and 15–25°C). The absolute temperature changes induced by stimulation are always around 2°C compared with control. We have described a physical mechanism that can underpin this correlation between the immune response role of macrophage cells and the lipid composition of their plasma membranes, where signalling activation initiates, as part of an amplification of response towards cell differentiation (to an activated, inflammed state). Moreover, for the first time, albeit in a preliminary fashion, we carried out experiments on vesicles reconstructed from purely the lipid fraction of GPMVs from the plasma membrane of macrophages. We observed their phase behaviour, comparing it with the GPMVs. The reconstructed vesicles show phase separation that is apparently the same as the GPMVs. The fact that separation is unaffected by the extraction of proteins means the lipids are undisputed key regulators of phase separation phenomena in the plasma membrane. Also for the first time, we quantified the fraction of irregular domains on GPMVs, which are a gel phase. We observed an increase of these at low temperatures. Much remains to be discovered within the ‘critical lipidomics’ paradigm; specifically, direct experiments are becoming possible owing to super-resolution approaches [4,11,12], probing membrane protein copy numbers and states of aggregation and how these are affected by their proximity to lipid mixture critical points.

## Supplementary Material

Supplementary Figures

## Data accessibility

Data are in the figures. Example datasets will be made available in an online repository (https://zenodo.org/record/3592489#.XjgVUmbgpaS).

## References

[1] Martinez FO, Gordon S 2014 The M1 and M2 paradigm of macrophage activation: time for reassessment. F1000Prime Rep. 6, 13 (10.12703/P6-13) 24669294 PMC3944738

[2] Taylor PR, Martinez-Pomares L, Stacey M, Lin HH, Brown GD, Gordon S 2005 Macrophage receptors and immune recognition. Annu. Rev. Immunol. 23, 901–944. (10.1146/annurev.immunol.23.021704.115816) 15771589

[3] Mosser DM, Edwards JP 2008 Exploring the full spectrum of macrophage activation. Nat. Rev. Immunol. 8, 958–969. (10.1038/nri2448) 19029990 PMC2724991

[4] Brandsma A *et al.* 2018 Mechanisms of inside-out signaling of the high-affinity IgG receptor Fc*γ*RI. Sci. Signal. 11, eaaq0891 (10.1126/scisignal.aaq0891) 30042128 PMC7521114

[5] Lawrence T, Natoli G 2011 Transcriptional regulation of macrophage polarization: enabling diversity with identity. Nat. Rev. Immunol. 11, 750–761. (10.1038/nri3088) 22025054

[6] Park BS, Song DH, Kim HM, Choi BS, Lee H, Lee JO 2009 The structural basis of lipopolysaccharide recognition by the TLR4-MD-2 complex. Nature 458, 1191–1195. (10.1038/nature07830) 19252480

[7] Kawai T, Akira S 2010 The role of pattern-recognition receptors in innate immunity: update on Toll-like receptors. Nat. Immunol. 11, 373–384. (10.1038/ni.1863) 20404851

[8] Akira S, Takeda K 2004 Toll-like receptor signalling. Nat. Rev. Immunol. 4, 499–511. (10.1038/nri1391) 15229469

[9] Simons K, Toomre D 2000 Lipid rafts and signal transduction. Nat. Rev. Mol. Cell Biol. 1, 31–39. (10.1038/35036052) 11413487

[10] Simons K, Sampaio JL 2011 Membrane organization and lipid rafts. Cold Spring Harb. Perspect. Biol. 3, a004697 (10.1101/cshperspect.a004697) 21628426 PMC3179338

[11] Stone MB, Shelby SA, Núñez MF, Wisser K, Veatch SL 2017 Protein sorting by lipid phase-like domains supports emergent signaling function in B lymphocyte plasma membranes. eLife 6, e19891 (10.7554/eLife.19891) 28145867 PMC5373823

[12] Veatch SL, Cicuta P 2018 *Critical lipidomics: the consequences of lipid miscibility in biological membranes*, pp. 141–168. Cham, Switzerland: Springer.

[13] Pralle A, Keller P, Florin EL, Simons K, Hörber JKH 2000 Sphingolipid-cholesterol rafts diffuse as small entities in the plasma membrane of mammalian cells. J. Cell Biol. 148, 997–1008. (10.1083/jcb.148.5.997) 10704449 PMC2174552

[14] Triantafilou M, Miyake K, Golenbock DT, Triantafilou K 2002 Mediators of innate immune recognition of bacteria concentrate in lipid rafts and facilitate lipopolysaccharide-induced cell activation. J. Cell Sci. 115, 2603–2611. 12045230 10.1242/jcs.115.12.2603

[15] Nakahira K *et al.* 2006 Carbon monoxide differentially inhibits TLR signaling pathways by regulating ROS-induced trafficking of TLRs to lipid rafts. J. Exp. Med. 203, 2377–2389. (10.1084/jem.20060845) 17000866 PMC2118097

[16] Wong SW, Kwon MJ, Choi AMK, Kim HP, Nakahira K, Hwang DH 2009 Fatty acids modulate toll-like receptor 4 activation through regulation of receptor dimerization and recruitment into lipid rafts in a reactive oxygen species-dependent manner. J. Biol. Chem. 284, 27384–27392. (10.1074/jbc.M109.044065) 19648648 PMC2785667

[17] Veatch SL, Cicuta P, Sengupta P, Honerkamp-Smith A, Holowka D, Baird B 2008 Critical fluctuations in plasma membrane vesicles. ACS Chem. Biol. 3, 287–293. (10.1021/cb800012x) 18484709

[18] Honerkamp-Smith A, Cicuta P, Collins MD, Veatch SL, Schick M, Keller SL 2008 Line tensions, correlation lengths, and critical exponents in lipid membranes near critical points. Biophys. J. 95, 236–246. (10.1529/biophysj.107.128421) 18424504 PMC2426649

[19] Scott RE 1976 Plasma membrane vesiculation: a new technique for isolation of plasma membranes. Science 194, 743–745. (10.1126/science.982044) 982044

[20] Scott RE, Maercklein PB 1979 Plasma membrane vesiculation in 3T3 and SV3T3 cells. II. Factors affecting the process of vesiculation. J. Cell Sci. 35, 245–252. 422673 10.1242/jcs.35.1.245

[21] Fridriksson EK, Shipkova PA, Sheets ED, Holowka D, Baird B, McLafferty FW 1999 Quantitative analysis of phospholipids in functionally important membrane domains from RBL-2H3 mast cells using tandem high-resolution mass spectrometry. Biochemistry 38, 8056–8063. (10.1021/bi9828324) 10387050

[22] Baumgart T, Hammond AT, Sengupta P, Hess ST, Holowka DA, Baird BA, Webb WW 2007 Large-scale fluid/fluid phase separation of proteins and lipids in giant plasma membrane vesicles. Proc. Natl Acad. Sci. USA 104, 3165–3170. (10.1073/pnas.0611357104) 17360623 PMC1805587

[23] Kaiser HJ, Lingwood D, Levental I, Sampaio JL, Kalvodova L, Rajendran L, Simons K 2009 Order of lipid phases in model and plasma membranes. Proc. Natl Acad. Sci. USA 106, 16 645–16 650. (10.1073/pnas.0908987106) PMC2757813 19805351

[24] Gray EM, Díaz-Vázquez G, Veatch SL 2015 Growth conditions and cell cycle phase modulate phase transition temperatures in RBL-2H3 derived plasma membrane vesicles. PLoS ONE 10, e0137741 (10.1371/journal.pone.0137741) 26368288 PMC4569273

[25] Burns M, Wisser K, Wu J, Levental I, Veatch S 2017 Miscibility transition temperature scales with growth temperature in a zebrafish cell line. Biophys. J. 113, 1212–1222. (10.1016/j.bpj.2017.04.052) 28552311 PMC5607031

[26] Tisza MJ, Zhao W, Fuentes JSR, Prijic S, Chen X, Levental I, Chang JT 2016 Motility and stem cell properties induced by the epithelial-mesenchymal transition require destabilization of lipid rafts. Oncotarget 7, 51553–51568. (10.18632/oncotarget.9928) 27303921 PMC5239496

[27] Dennis EA *et al.* 2010 A mouse macrophage lipidome. J. Biol. Chem. 285, 39976–39985. (10.1074/jbc.M110.182915) 20923771 PMC3000979

[28] Andreyev AY *et al.* 2010 Subcellular organelle lipidomics in TLR-4-activated macrophages. J. Lipid Res. 51, 2785–2797. (10.1194/jlr.M008748) 20574076 PMC2918461

[29] Hoshino K, Takeuchi O, Kawai T, Sanjo H, Ogawa T, Takeda Y, Takeda K, Akira S 1999 Cutting edge: toll-like receptor 4 (TLR4)-deficient mice are hyporesponsive to lipopolysaccharide: evidence for TLR4 as the Lps gene product. J. Immunol. 162, 3749–3752. 10201887

[30] Vats D, Mukundan L, Odegaard JI, Zhang L, Smith KL, Morel CR, Greaves DR, Murray PJ, Chawla A 2006 Oxidative metabolism and PGC-1*β* attenuate macrophage-mediated inflammation. Cell Metab. 4, 13–24. (10.1016/j.cmet.2006.05.011) 16814729 PMC1904486

[31] Tatano Y, Shimizu T, Tomioka H 2014 Unique macrophages different from M1/M2 macrophages inhibit T cell mitogenesis while upregulating Th17 polarization. Sci. Rep. 4, 4146 (10.1038/srep04146) 24553452 PMC3930092

[32] Kigerl KA, Gensel JC, Ankeny DP, Alexander JK, Donnelly DJ, Popovich PG 2009 Identification of two distinct macrophage subsets with divergent effects causing either neurotoxicity or regeneration in the injured mouse spinal cord. J. Neurosci. 29, 13 435–13 444. (10.1523/JNEUROSCI.3257-09.2009) PMC2788152 19864556

[33] Sezgin E, Kaiser HJ, Baumgart T, Schwille P, Simons K, Levental I 2012 Elucidating membrane structure and protein behavior using giant plasma membrane vesicles. Nat. Protoc. 7, 1042–1051. (10.1038/nprot.2012.059) 22555243

[34] Gray E, Karslake J, Machta BB, Veatch SL 2013 Liquid general anesthetics lower critical temperatures in plasma membrane vesicles. Biophys. J. 105, 2751–2759. (10.1016/j.bpj.2013.11.005) 24359747 PMC3882514

[35] Bligh EG, Dyer WJ 1959 A rapid method of total lipid extraction and purification. Can. J. Biochem. Physiol. 37, 911–917. (10.1139/y59-099) 13671378

[36] Weinberger A, Tsai F, Koenderink G, Schmidt T, Itri R, Meier W, Schmatko T, Schröder A, Marques C 2013 Gel-assisted formation of giant unilamellar vesicles. Biophys. J. 105, 154–164. (10.1016/j.bpj.2013.05.024) 23823234 PMC3699747

[37] Sakai J, Cammarota E, Wright JA, Cicuta P, Gottschalk RA, Li N, Fraser IDC, Bryant CE 2017 Lipopolysaccharide-induced NF-*κ*B nuclear translocation is primarily dependent on MyD88, but TNF*α* expression requires TRIF and MyD88. Sci. Rep. 7, 1428 (10.1038/s41598-017-01600-y) 28469251 PMC5431130

[38] Vasan M, Wolfert MA, Boons GJ 2007 Agonistic and antagonistic properties of a rhizobium sin-1 lipid A modified by an ether-linked lipid. Org. Biomol. Chem. 5, 2087–2097. (10.1039/b704427e) 17581652 PMC2830616

[39] Daniel B *et al.* 2018 The nuclear receptor PPAR*γ*B controls progressive macrophage polarization as a ligand-insensitive epigenomic ratchet of transcriptional memory. Immunity 49, 615–626. (10.1016/j.immuni.2018.09.005) 30332629 PMC6197058

[40] Levental KR, Surma MA, Skinkle AD, Lorent JH, Zhou Y, Klose C, Chang JT, Hancock JF, Levental I 2017 *ω*-3 polyunsaturated fatty acids direct differentiation of the membrane phenotype in mesenchymal stem cells to potentiate osteogenesis. Sci. Adv. 3, eaao1193 (10.1126/sciadv.aao1193) 29134198 PMC5677358

[41] Stow JL, Ching Low P, Offenhäuser C, Sangermani D 2009 Cytokine secretion in macrophages and other cells: pathways and mediators. Immunobiology 214, 601–612. (10.1016/j.imbio.2008.11.005) 19268389

[42] Fortes FSA *et al.* 2004 Modulation of intercellular communication in macrophages: possible interactions between GAP junctions and P2 receptors. J. Cell Sci. 117, 4717–4726. (10.1242/jcs.01345) 15331634

[43] Lim TS, Mortellaro A, Lim CT, Hämmerling GJ, Ricciardi-Castagnoli P 2011 Mechanical interactions between dendritic cells and T cells correlate with T cell responsiveness. J. Immunol. 187, 258–265. (10.4049/jimmunol.1100267) 21622857

[44] Ayuyan A, Cohen FS 2018 The chemical potential of plasma membrane cholesterol: implications for cell biology. Biophys. J. 114, 904–918. (10.1016/j.bpj.2017.12.042) 29490250 PMC5984996

[45] Dietrich C, Bagatolli LA, Volovyk ZN, Thompson NL, Levi M, Jacobson K, Gratton E 2001 Lipid rafts reconstituted in model membranes. Biophys. J. 80, 1417–1428. (10.1016/S0006-3495(01)76114-0) 11222302 PMC1301333

[46] Kimchi O, Veatch S, Machta BB 2018 Ion channels can be allosterically regulated by membrane domains near a de-mixing critical point. J. Gen. Physiol. 150, 1769–1777. (10.1085/jgp.201711900) 30455180 PMC6279359

[47] Bryant CE, Spring DR, Gangloff M, Gay NJ 2010 The molecular basis of the host response to lipopolysaccharide. Nat. Rev. Microbiol. 8, 8–14. (10.1038/nrmicro2266) 19946286

[48] Sung MH, Li N, Lao Q, Gottschalk RA, Hager GL, Fraser IDC 2014 Switching of the relative dominance between feedback mechanisms in lipopolysaccharide-induced NF-*κ*B signaling. Sci. Signal. 7, 1–11. (10.1126/scisignal.2004764) PMC5381725 24425788

[49] Płóciennikowska A, Hromada-Judycka A, Borzęcka K, Kwiatkowska K 2014 Co-operation of TLR4 and raft proteins in LPS-induced pro-inflammatory signaling. Cell. Mol. Life Sci. 72, 557–581. (10.1007/s00018-014-1762-5) 25332099 PMC4293489

[50] Pfeiffer A *et al.* 2001 Lipopolysaccharide and ceramide docking to CD14 provokes ligand-specific receptor clustering in rafts. Eur. J. Immunol. 31, 3153–3164. (10.1002/1521-4141(200111)31:11<3153::aid-immu3153>3.0.co;2-0) 11745332

[51] Triantafilou M, Brandenburg K, Kusumoto S, Fukase K, Mackie A, Seydel U, Triantafilou K 2004 Combinational clustering of receptors following stimulation by bacterial products determines lipopolysaccharide responses. Biochem. J. 381, 527–536. (10.1042/BJ20040172) 15040785 PMC1133861

[52] Matsumura H, Nakano M 1988 Endotoxin-induced interferon-gamma production in culture cells derived from BCG-infected C3H/HeJ mice. J. Immunol. 140, 494–500. 3121747

[53] Darmani H, Parton J, Harwood JL, Jackson SK 1994 Interferon-gamma and polyunsaturated fatty acids increase the binding of lipopolysaccharide to macrophages. Int. J. Exp. Pathol. 75, 363–368. 7999637 PMC2001866

[54] Netea MG, Joosten LAB, Latz E, Mills KHG, Natoli G, Stunnenberg HG, O’Neill LAJ, Xavier RJ 2016 Trained immunity: a program of innate immune memory in health and disease. Science 352, aaf1098 (10.1126/science.aaf1098) 27102489 PMC5087274

[55] Frechin M, Stoeger T, Daetwyler S, Gehin C, Battich N, Damm EM, Stergiou L, Riezman H, Pelkmans L 2015 Cell-intrinsic adaptation of lipid composition to local crowding drives social behaviour. Nature 523, 88–91. (10.1038/nature14429) 26009010

[56] Lee CM, Hu J 2013 Cell density during differentiation can alter the phenotype of bone marrow-derived macrophages. Cell Biosci. 3, 30 (10.1186/2045-3701-3-30) 23895502 PMC3750618

