# Supplementary material for: Criticality of plasma membrane lipids reflects activation state of macrophage cells
Source: J R Soc Interface. 2020 Feb 5;17(163):20190803. doi: 10.1098/rsif.2019.0803 (PMC7061703; doi:10.1098/rsif.2019.0803)
Supplement: Supplementary Figures [file rsif20190803supp1.pdf]

royalsocietypublishing.org

Research

Article submitted to journal

Subject Areas:

xxxxx, xxxxx, xxxx

Keywords:

liquid liquid phase separation; plasma membrane composition; critical lipidomics

Author for correspondence:

Pietro Cicuta

e-mail: pc245@cam.ac.uk

Supplementary Material of: Criticality of plasma membrane lipids reflects activation state of macrophage cells

Eugenia Cammarota[1,2], Chiara Soriani[1], Raphaelle Taub[1], Fiona Morgan[1], Jiro Sakai[3], Sarah L. Veatch[4], Clare E. Bryant[3], Pietro Cicuta[1]

[1]Cavendish Laboratory, University of Cambridge, JJ Thomson Av., Cambridge CB3 0HE, UK

[2]Experimental Imaging Center, San Raffaele Hospital, Milan, Italy

[3]Department of Veterinary Medicine, University of Cambridge, Cambridge CB3 0ES, United Kingdom

[4]Biophysics Department, University of Michigan, Ann Arbor, MI 48109, USA

Supplementary Materials

THE ROYAL SOCIETY PUBLISHING

© The Author(s) Published by the Royal Society. All rights reserved.

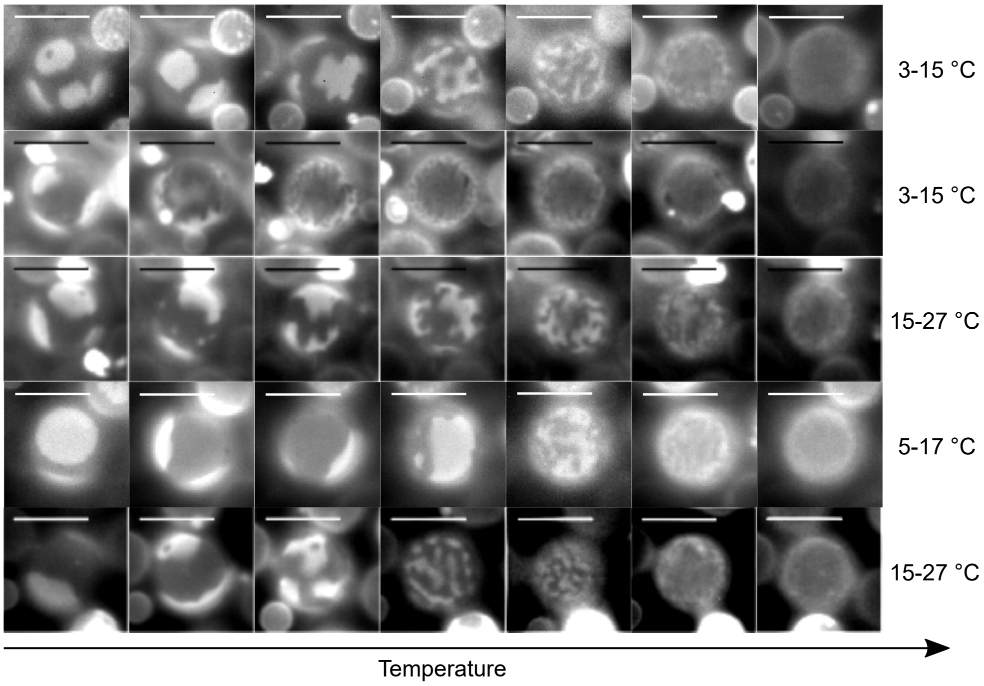

Figure S1. Images of 5 different GPMVs undergoing phase transition. In this sequence images are acquired increasing the temperature at steps of 2°C starting from below the melting temperature. In systems with critical composition, approaching the critical temperature, the line tension between separate phases progressively decreases up until the appearance of a unique phase. In vesicles with critical lipid composition this causes the appearance of rough borders around the L_o/l_d phase separation domains until the two phases are indistinguishable. GPMVs from macrophages look to have a similar behaviour suggesting a lipid composition close to critical. Scalebar 2μm. In each image the contrast has been indipendently adjusted to better visualize the domain's shape.

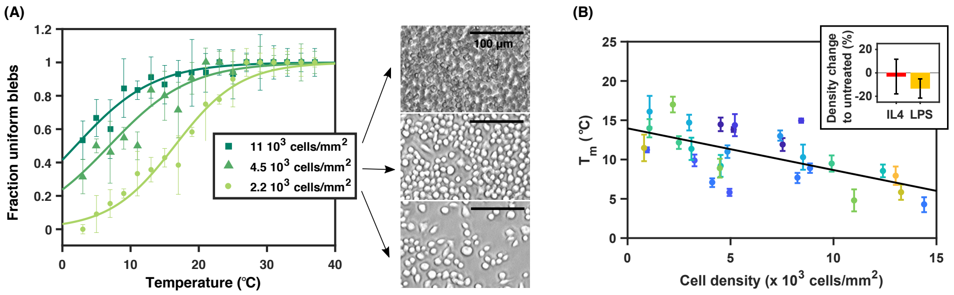

Figure S2. Cell crowding affects the phase transition of GPMVs. We report here the results of experiments done growing cells in a common flask, and then plated at different densities. (A) There is a consistent shift in the data for the fraction of uniform GPMVs, from cell cultures at different densities so that the curve that corresponds to the most crowded sample is on the right of the less dense samples. T_{11} = (4.8 $\pm$ 1.4)$^\circ$C, $T_{4.5}$ = (8.9 $\pm$ 1.2)$^\circ$C, $T_{2.2}$ = (17.0 $\pm$ 1.0)$^\circ$C. (B) The miscibility temperatures obtained from the sigmoidal curve fitting, as a function of the cell crowding, showing that the denser samples have lower transition temperature. Same colors indicate repetition of the experiment on same day. The linear fit $y = a + cx$ gives c = (-0.53 $\pm$ 0.26)$^\circ$C mm^2/cells and a = (14.0 $\pm$ 1.9)$^\circ$C. We had at this point to check the possibility that the temperature shift observed as a function of the pro/anti-activation treatment might be an indirect effect, due to a differential stimulus-dependent growth. To check for this, we measured cell growth through the difference in cell density after IL4 and LPS stimulation. In the sub-panel is represented the density change as effect of 12 hours of stimulation compared to an untreated sample. Reproducing our typical experimental conditions (7$\cdot$10^3 cells/mm^2, and 12 h stimulation), we obtained a non-significant change in the density of IL4 treated cells compared to the unstimulated condition (-3 $\pm$ 15)% , while the LPS showed a decrease of (-13 $\pm$ 8)% (values obtained over 4 repetitions). Putting together the growth rate reduction with LPS with the calibrated cell-concentration results, for the LPS condition we obtain (as an indirect effect of the stimulant on the cell culture growth rate) an expected change of the melting temperature of about -0.5°C compared to the untreated condition. Therefore this important control shows that the ~ 2 degrees difference in T_m seen between untreated and LPS stimulated is due only in small part to cell density, so most of the effect has to be accounted for by processes independent of density, downstream of the LPS signalling pathway.

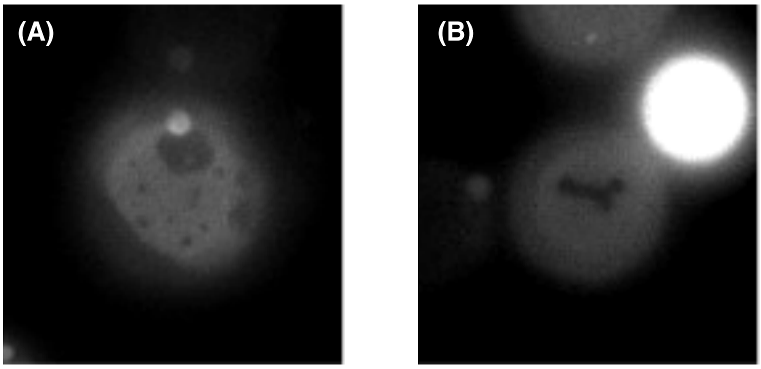

Figure S3. Example GPMVs showing round domains (A) and an irregular domain (B). These different shapes likely correspond respectively to liquid-liquid and liquid-gel phase coexistence.

royalsocietypublishing.org 0000000

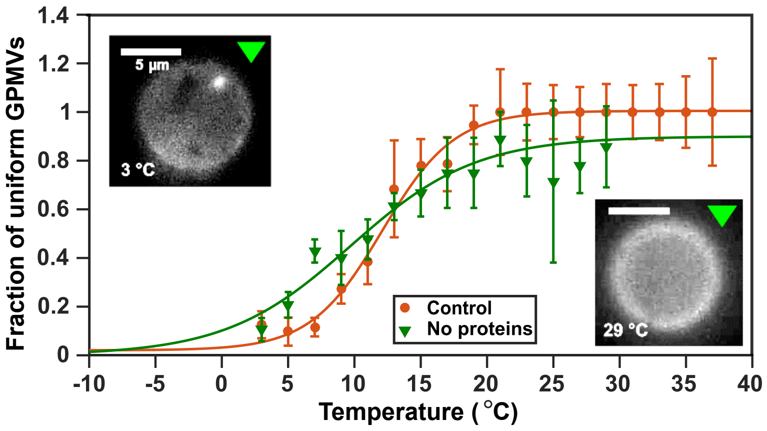

Figure S4. A key control with purified lipids excludes the role of proteins in the phenomena reported above. Comparison between the distribution of uniform GPMVs against analysis of vesicles re-formed from the just the lipidic component purified from the same sample. The melting temperature is compatible, within the error, meaning that the lipids are unperturbed in the determination of the melting temperature. $T_{noproteins}$ = (11.1$\pm$1.4)°C, $T_{control}$ = (12.2$\pm$0.5)°C. See also Supplementary Figure S7.

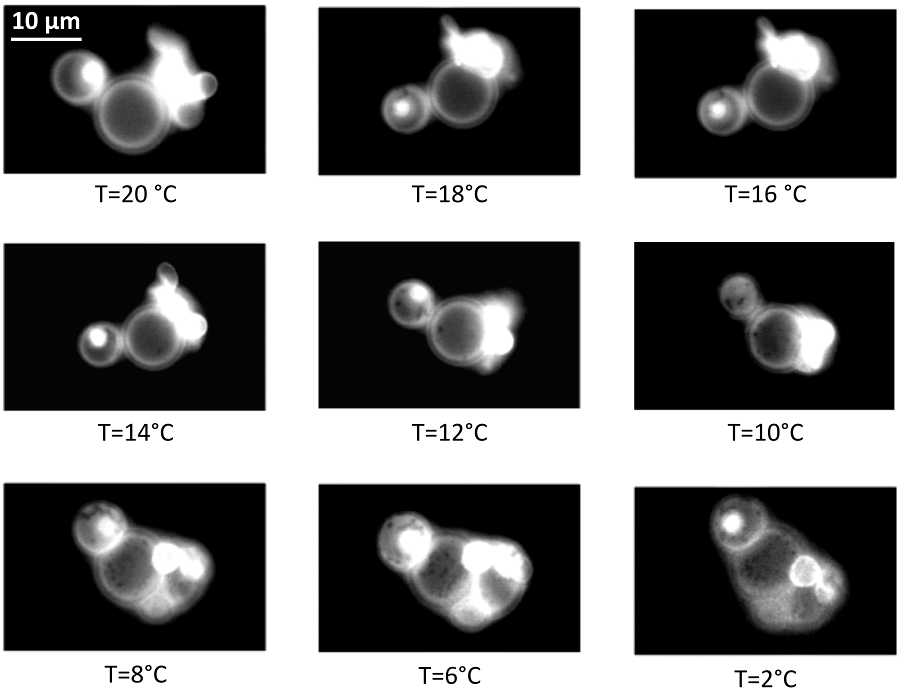

Figure S5. In the same way as the 'parent' GPMV, it has been checked that vesicles reformed from just the lipid fraction purified from GPMVs undergo phase separation. The images show phase separation (dark domains) appearing at 18 and 12°C.

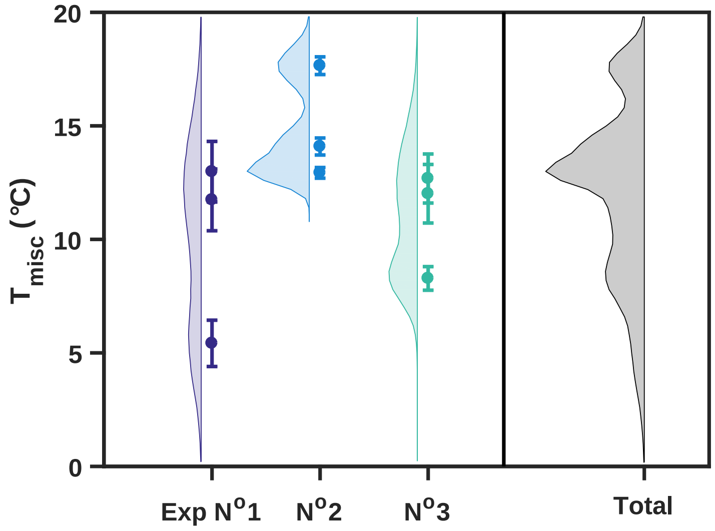

Figure S6. Miscibility temperature of three repetitions of the same experiment in which macrophage cells at the same density are cultured in three separate flasks as replicates for two days. The data points have a wide temperature range both within the replicates and comparing the three experiments. Data points are also interpreted as representative of a whole distribution with the same average and standard deviation. In spite of the large variation, the sum of the distributions shows a main peak around 13°C. Continuous distributions are obtained simulating gaussian distributed numbers with mean and standard deviation equal to the value of the data and their error.

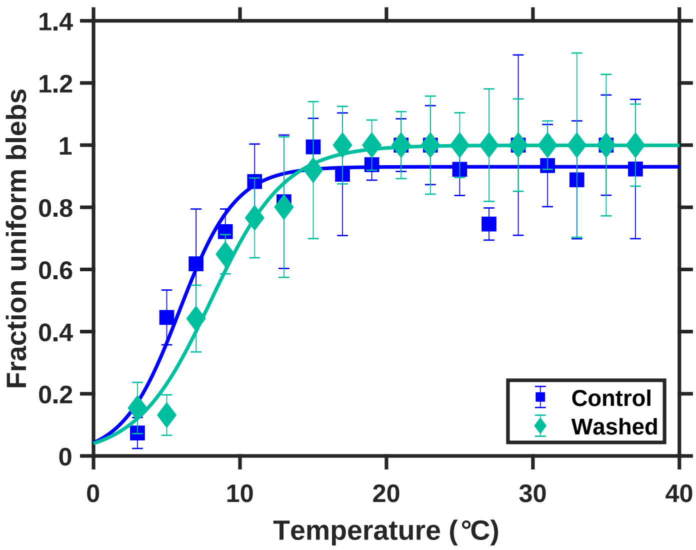

Figure S7. Control experiment to test the effect of intracellular communication through secretion of cytokines on the melting temperature of GPMVS. The two samples where plated at the same density, and blebbing was induced after 12 hours during which the medium of the "washed" sample was changed every two hours. The control sample shows a lower T_m compared to the "washed". This is compatible with a scenario in which the control condition is affected by un accumulation of M2-inducing cytokines like IL4. T_{contol} = 5.8 $\pm$ 1.0, T_{washed} = 7.9 $\pm$ 1.2.
